# The Urinary Concentration of Trefoil Factor 3 (TFF3) in the Term and Preterm Neonates

**DOI:** 10.3390/jcm12154936

**Published:** 2023-07-27

**Authors:** Monika Kamianowska, Agnieszka Rybi-Szumińska, Aleksandra Kamianowska, Mateusz Maciejczyk, Katarzyna Sołomianko, Alicja Koput, Anna Wasilewska

**Affiliations:** 1 Department of Neonatology and Neonatal Intensive Care, Medical University of Bialystok, M. C. Sklodowskiej 24a Street, 15-276 Białystok, Poland; slomkasia@o2.pl; 2 Department of Pediatrics and Nephrology, Medical University of Bialystok, 15-269 Bialystok, Polandolcikkam@wp.pl (A.K.);; 3 Department of Hygiene, Epidemiology and Ergonomic, Medical University of Bialystok, 15-269 Bialystok, Poland; 4 Department of Pediatric Laboratory Diagnostics, Medical University of Bialystok, 15-269 Bialystok, Poland

**Keywords:** Trefoil factor 3, biomarkers, kidney, neonates, kidney disease

## Abstract

Background: Distinguishing between a pathologic state and renal development is important in neonatology. Because the assessment of serum creatinine in neonates is not reliable, better biomarkers are needed. Trefoil factor 3 (TFF3) is proposed as a biomarker of kidney injury. The study aimed to assess its urinary concentration in healthy term and stable preterm neonates. Material and methods: The study included 80 term and 20 preterm neonates born in the Department of Perinatology of the University Clinical Hospital in Bialystok. Urine was obtained from the term neonates on the 1st day of life and from the preterm neonates on the 1st, 8th, 15th and 22nd day of life. The urinary concentration of TFF3 was determined using a commercially available immunoassay and was normalized for the urinary creatinine concentration (cr.). Results: The values of TFF3/cr. were higher in the preterm than in the term neonates (*p* < 0.05) (median (Q1–Q3): 1486.85 (614.92–3559.18) and 317.29 (68.07–671.40) ng/mg cr.). They did not differ in the subsequent days of the preterm neonates’ lives. The ROC curve for TFF3/cr. in the preterm and term neonates showed AUC = 0.751 (cut-off value = 1684.25 ng/mg cr.). Conclusions: Prematurity is associated with higher urinary excretion of TFF3. Male gender is associated with an increased urinary TFF3 excretion in term neonates.

## 1. Introduction

The prematurity of neonates’ kidneys, especially in preterm neonates, whose kidneys are in an active nephrogenesis, makes them especially prone to the negative effects of the extra-uterine environment. These effects may lead to their failure [1]. Only early diagnosis and treatment offer a chance to restore the normal kidney function. However, the diagnostic process in the neonatal period is difficult due to the unreliability of the main marker of renal function—the serum creatinine concentration—in this age group. This is a very late indicator of kidney damage (changes in serum creatinine concentrations can be observed only when more than 50% of the kidney is not functioning properly) and depends on a number of factors, including the clinical condition of the mother [2].

Similarly, it is difficult to answer the diagnostic question: is it a pathology or is it still an adaptation. The process of nephrogenesis is complete in full-term neonates, but functional adaptive changes continue as the infant matures [3]. However, because over 60% of nephrons are formed in the 3rd trimester of pregnancy, in preterm neonates, the nephrogenesis is not completed at the time of delivery and continues during the first 40 days of life [4,5,6,7]. Searching for a new biomarker of the renal functions and trying to answer the above-mentioned question, we first included neonates in the adaptation period without signs of kidney damage in the study. Studies on healthy controls are essential to clearly understand the biomarkers’ physiology and to use them in practice [8].

Many biomarkers of kidney injury, such as NGAL, KIM-1, Cystatin C and others, have already been studied. However, many candidate biomarkers did not prove their sufficient sensitivity and specificity for routine clinical use. The possible explanation of this phenomenon is the insufficient understanding of their biological role. In this case, new potential biomarkers are studied to eventually find one with good clinical sensitivity and specificity and to improve the prediction, detection and diagnosis of kidney injury [9]. One of them is Trefoil Factor 3 (TFF3).

TFF3, a member of the trefoil factor family, is a small peptide (molecular weight: 7 kDa) secreted by cells producing mucous and involved in both the maintenance and repair of the mucosal surface [10]. TFF3 is secreted by epithelial cells, including the epithelial cells of the renal tubules [11]. Immunohistochemistry shows that it is expressed in the tubular cells in the kidney’s cortex. No expression of TFF3 is found in the glomeruli, interstitium and arterioles [12]. TFF3 has a role in both the restitution and regeneration of the epithelia. It promotes the migration of the cells to the lesions and their differentiation and proliferation as a part of repair [13]. Because of its functions in the urinary tract epithelium, it was proposed as a urine biomarker for kidney damage [14]. As it takes part in both the restitution and regeneration of the epithelia, it seems reasonable that it could indicate the ongoing injury of the epithelium [14]. Moreover, it is secreted only by tubular cells in the kidney’s cortex and not in the glomerulus, interstitium and arterioles. As a result, its elevated amounts in urine could indicate the injury exactly in the proximal and distal convoluted tubules. This phenomenon could be the basis for the effective localization of the site of kidney damage [12].

The aim of this study was to analyze the excretion of the TFF3 after birth in healthy full-term and stable preterm infants, and to analyze the fluctuations of the TFF3 excretion during the first weeks of the neonates’ lives.

## 2. Patients and Methods

### 2.1. Patient Recruitment

This prospective study was conducted in the Department of Neonatology and Neonatal Intensive Care at the Medical University of Bialystok. The study was conducted according to the guidelines of the Declaration of Helsinki. The study protocol was approved by the Local Bioethics Committee of the Medical University of Bialystok (date of approval: 16 December 2021, protocol code: APK.002.502.2021). Written, informed consents were obtained from the parents of all the neonates prior to the study. The parents were informed of the aim, the method and the proceedings after receiving the results.

Overall, 100 neonates were included in the study. The neonates were born in the Department of Perinatology and then hospitalized in the Department of Neonatology and Neonatal Intensive Care of the University Clinical Hospital in Bialystok between 1 January 2022 and 31 January 2023.

The children were divided into 2 groups: the group comprising 80 term neonates (born between 37 weeks and 42 weeks of pregnancy) and the group comprising 20 preterm neonates (born between 23 and completed 36 weeks of pregnancy). The aim of the study was the assessment of the values of TFF3 in healthy newborns; strict inclusion criteria were used. All prenatal and postnatal factors that might influence the function of the kidneys had to be excluded. The main inclusion criteria were: birth in the Department of Perinatology of the University Clinical Hospital in Bialystok, the parents’ written consent, appropriate gestational age (23–36 week of pregnancy for preterm neonates and 37–42 weeks of pregnancy of term neonates).

The general exclusion criteria concerning the neonatal factors were congenital abnormalities, genetic disorders and congenital infections. The general exclusion criteria concerning the perinatal factors were abnormal prenatal examination. The general exclusion criteria concerning maternal factors were diseases during the pregnancy (infections, disorders of metabolism, cardiological, pulmonary, hematological, systemic and endocrine disorders and other disorders requiring the supply of drugs)

The specific exclusion criteria in the group of neonates born between 23 and 36 weeks of pregnancy (the examined group) were: 1st minute Apgar score < 4, severe general condition, mechanical ventilation, abnormal ultrasound examination of the central nervous system (the following abnormalities were accepted: 1st degree intraventricular bleeding, hyper-echoic zones around the lateral ventricles), abnormal ultrasound examination of the abdominal cavity, urinary tract defects (hydronephrosis, polycystic kidney disease, duplex kidney, duplex ureters, agenesis of the kidney, other anatomical abnormalities), intrauterine growth retardation, abnormalities in the laboratory tests (abnormal morphology and biochemistry), administration of drugs other than mandatory (vaccination against hepatitis B (Euvax B, LG Life Sciences, Warsaw, Poland), against tuberculosis (BCG, Biomed Lublin SA, Lublin, Poland), vitamin K (Kanavit, BB Pharma, Praha, The Czech Republic or Konakion, Prima Infanzia, Roche Pharma AG, Basel, Switzerland), vitamin D3 (Devikap, Polpharma, Starogard Gdanski, Poland) and other than parenteral nutrition (Numeta G13 %E, Baxter, Warsaw, Poland), methylxanthines (Peyona, Chiesi Farmaceutici, Parma, Italy), supplementation of micro and macro elements under the standards, exposure to nephrotoxic agents.

The specific exclusion criteria in the group of neonates born between 37 and 42 weeks of pregnancy (the reference group) were: 1st minute Apgar score < 7, average and severe general condition, administration of drugs other than mandatory (vaccination against hepatitis B (Euvax B, LG Life Sciences, Poland), against tuberculosis (BCG, Biomed Lublin SA, Poland), vitamin K (Kanavit, BB Pharma, The Czech Republic or Konakion Prima Infanzia, Roche Pharma AG, Schweiz), vitamin D3 (Devikap Polpharma, Poland)).

The conditions for terminating the study were: nosocomial infection of the neonate, deterioration of the general condition, withdrawal of the parental consent for the study.

**Figure 1:**
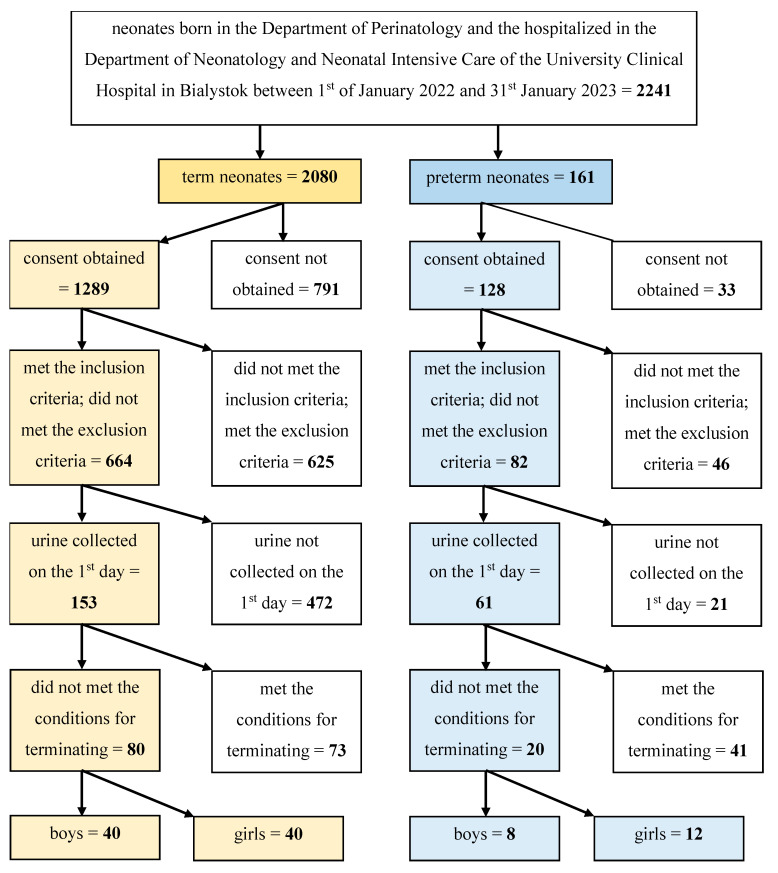
The algorithm of patients’ screening and creation of the final number of patients included in the study.

The algorithm of the patients’ screening and the creation of the final number of patients included in the study are presented in the Figure 1.

### 2.2. Sample Collection

The samples of the term neonates’ urine were collected once on the 1st day of life. The samples of the preterm neonates were collected four times, on the 1st, 8th, 15th and 22nd day of life. The collection of the urine was conducted noninvasively, using single-use, sterile bags (ZARYS, Zabrze, Poland). The centrifugate urine samples were kept in the refrigerator at 4 °C; then, after no more than 2 h, they were frozen at −80 °C. The samples were stored at −80 °C for no longer than 4 months and were not frozen and thawed multiple times.

The samples of the preterm neonates’ blood were collected during the 1st or 2nd day of the child’s life to S-Monovette 1.2 mL, Clotting Activator/Serum test tubes (Sarstedt AG & Co., Nümbrecht, Germany) for venous blood. The collection of venous blood was part of a routine practice in the department. The blood morphology and biochemistry tests were conducted immediately after obtaining the samples.

### 2.3. Determination of Basic Blood and Urine Parameters

The estimated GFR (eGFR) was calculated using the Schwartz formula appropriate for preterm neonates ($eGFR=\frac{0.33\cdot length\;}{sCr}$, where L—length in centimeters (cm), Scr—concentration of serum creatinine in milligrams per deciliter (mg/dL)) and presented in milliliters per minute per 1.73 square meter (mL/min/1.73 m^2^).

The concentrations of creatinine in the serum and urine were measured using Jaffé’s method and expressed in milligrams per deciliter (mg/dL).

The blood morphology and biochemistry tests were performed in the Department of Laboratory Diagnostics at the University Clinical Hospital in Bialystok during the routine practice in the laboratory. The blood morphology tests were conducted using the Sysmex xn-1500 analyzer (Sysmex Corporation, Kobe, Japan). The blood biochemistry tests were conducted using the Abbott Alinity analyzer (Abbott Laboratories, Abbott Park, IL, USA).

### 2.4. Determination of the Concentration of TFF3 and the Value of TFF3/cr

The concentrations of TFF3 in the urine were measured using a commercially available immunoassay for kidney toxicology research—Bio-Plex Pro™ RBM Human Kidney Toxicity Panel 2 (BIO-RAD, Hercules, CA, USA). The assay’s working range was (lower limit of quantification (LLOQ)-upper limit of quantification (ULOQ)): 0.075–98 ng/mL. The assay’s sensitivity (limit of detection, LOD) was 0.036 ng/mL. The intra-assay and inter-assay coefficients of variation (%CV) were, respectively, 2% and 6%. The concentration of TFF3 was expressed in nanograms per milliliter (ng/mL).

Because urinary dilution may have a potentially confounding effect, the concentration of TFF3 was normalized for the urinary concentration of creatinine. The TFF3/cr. ratio was expressed in nanograms per milligram of creatinine (ng/mg cr.).

The determination of the concentration of TFF3 was conducted in the Department of Hygiene, Epidemiology and Ergonomics of the Medical University of Bialystok.

### 2.5. Statistical Analysis

The statistical analysis of the data was performed using the Statistica 13.3 package (StatSoft, Cracow, Poland). The discrete variables were expressed as counts (percentage, %). The continuous variables were expressed as median, 1st quartile and 3rd quartile (Median (Q1–Q3)). The type of distribution was checked using the Shapiro-Wilk test. Because the data were not normally distributed, the intergroup comparison of the continuous variables was performed using the Mann-Whitney U test. To determine whether the proportions of one nominal variable were different depending on the value of the other nominal variable, the Fisher’s exact test of independence was used. The correlations between both the concentration of TFF3 and the value of the TFF3/cr. ratio, and other variables, were established using the Spearman’s rank correlation coefficients. The direction and power of the associations were determined. The comparison of the values of the two dependent groups was performed using the Wilcoxon signed-rank test. The value of the significance level was α = 0.05. The results were statistically significant when *p* < 0.05.

In the group of term neonates, a 95% confidence interval was determined in order to estimate the range of values likely to include a population value with a 95% confidence.

The ROC curve (receiver operating characteristic) and the AUC (area under the ROC curve) were used to assess the capability of TFF3 to distinguish between term and preterm neonates (normal and impaired renal function). The following parameters were also calculated for the TFF3/cr ratio: the sensitivity and specificity of the test, the positive and negative predictive values (PPV and NPV, respectively)

There were some missing data on the parameters of blood biochemistry. The missing data were classified as MCAR (missing completely at random) and replaced with the average value calculated based on the known values (Mean Imputation). There were no missing data for the concentration of TFF3 and the value of the TFF3/cr. ratio.

## 3. Results

100 healthy neonates were included in the study. The group comprised 80 healthy term neonates and 20 stable preterm neonates. In total, 40 boys and 40 girls formed the group of term neonates. The group of preterm children comprised 8 boys and 12 girls. Both groups were sex-matched (*p* > 0.05).

### 3.1. Characteristics of the Term Neonates

All of the term neonates were appropriate for gestational age (AGA) and in good condition (1st minute Apgar score ≥ 8). No significant differences were found between the term boys and girls in the following parameters: gestational age, type of delivery, birth weight, length and head circuit.

### 3.2. Characteristics of the Preterm Neonates

Table 1 presents the characteristics of the preterm neonates included in the study.

The analysis of the basic characteristics of the preterm neonates showed that the gestational age of the boys was significantly higher than the gestational age of the girls (*p* < 0.05). The proportion of vaginal-born and cesarean-born children did not differ between the male and female neonates (*p* > 0.05). The predominance of cesarean-born children was found in both groups. The female preterm children had significantly lower birth weights than the male neonates (*p* < 0.05). The length and head circuit did not differ between the boys and girls (*p* > 0.05).

The group of preterm neonates was divided into two subgroups based on the children’s gestational age: very preterm neonates (born at less than 32 weeks of pregnancy) and moderately and late preterm neonates (born between 32 and 36 completed weeks of pregnancy). Very preterm children accounted for 65.00% (*N* = 13) of the preterm children and moderately or late preterm children accounted for 35.55% (*N* = 5). The proportion of the above-mentioned groups did not differ between the boys and girls (*p* < 0.05).

The group of preterm neonates was also divided into two subgroups based on the children’s birth weight: low birth weight neonates (LBW, 1500 to 2500 g) and very low birth weight neonates (VLBW, 1000 to 1499 g). One girl weighed 840 g and was classified as an extremely low birth weight neonate (ELBW). The LBW children accounted for 55.00% of all the preterm neonates. The proportion of the above-mentioned groups differed between the boys and girls (*p* < 0.05). A significantly higher ratio of VLBW/LBW characterized the group of female children.

No significant difference between the 1st, 3rd, 5th and 10th minute Apgar scores was found between the boys and girls.

The basic laboratory tests were performed in the group of preterm neonates. We assessed the blood morphology (leukocytes, hemoglobin, hematocrit and platelets) and the blood biochemistry (C-reactive protein, procalcitonin, interleukin-6, urea, aspartate aminotransferase, alanine aminotransferase, bilirubin, protein, sodium, potassium, calcium, magnesium, phosphorus). The analysis of the above-mentioned tests did not show any significant differences between the boys and girls. All of the results were within the normal range, according to the reference values of the Department of Laboratory Diagnostics at the University Clinical Hospital in Bialystok.

All of the preterm neonates had normal parameters of renal function (serum and urinary concentrations of creatinine, eGFR). The concentrations of the serum and urine creatinine did not differ between the boys and girls (*p* > 0.05). Also, the eGFR did not show a significant difference between the male and female neonates (*p* > 0.05).

### 3.3. The Analysis of the Concentration of TFF3 and the Value of TFF3/cr. Ratio on the 1st Day of Life

Table 2 shows the concentrations of TFF3 and the values of the TFF3/cr. ratio on the 1st day of life.

The statistical analysis showed that in the group of term neonates, both the concentrations of TFF3 in the urine and the values of the TFF3/cr. ratio were significantly higher in the boys than in the girls (*p* < 0.01 in both cases). In the group of preterm neonates, no significant differences in the above-mentioned parameters were found between the boys and girls (*p* > 0.05 in both cases).

The concentrations of TFF3 and the values of the TFF/cr. ratio were compared between the term and preterm neonates. In the groups of both male and female children, the concentrations of TFF3 did not differ between the term and the preterm neonates. However, the values of the TFF3/cr. ratio were significantly higher in the group comprising the preterm neonates.

In the group of term and preterm neonates, no statistically significant differences in the concentration of TFF3 and the value of the TFF3/cr. ratio were found between cesarean delivery and vaginal-born children.

The concentrations of TFF3 and the values of the TFF/cr. ratio were compared between the neonates with different body weights. In the group comprising the term neonates, no significant differences were found in these parameters between the children born with a body weight between the 10th and 50th percentile and between the 51st and 90th percentile (*p* > 0.05 in both cases). In the group comprising preterm neonates, no significant differences in the above-mentioned parameters were found between the LBW and VLBW neonates (*p* > 0.05 in both cases)

The concentrations of TFF3 and the values of the TFF/cr. ratio were compared between the preterm neonates with different gestational ages. No significant differences in the above-mentioned parameters were found between the very preterm and moderately + late preterm neonates (*p* > 0.05 in both cases).

The 95% confidence intervals (CI) for the mean of a normal population were estimated in the group of term neonates. In addition, 95% CI for the concentration of TFF was 229.15–427.88 ng/mL and the 95% CI for the value of the TFF3/cr. ratio was 436.33–1079.67 ng/mg cr.

### 3.4. The Analysis of the Concentration of TFF3 and the Value of TFF3/cr. Ratio Correlations with Other Parameters on the 1st Day of Life

In the group of term neonates, a negative correlation was found between the value of the TFF3/cr. ratio and the gestational age (R = −0.30, *p* < 0.01). No significant correlations were found between the concentration of TFF3 and the other examined variables.

In the group of preterm neonates, a positive correlation was found between the concentration of TFF3 and the platelet count in the blood (R = 0.58, *p* < 0.01) and the concentration of potassium in the plasma (R = 0.46, *p* < 0.05). Also, the value of TFF3/cr. correlated positively with the platelet count (R = 0.48, *p* < 0.05).

### 3.5. The Analysis of the Concentrations of TFF3 and the Values of TFF3/cr. Ratio on the 1st, 8th, 15th and 22nd Day of Life

Table 3 shows the concentrations of TFF3 and the values of the TFF3/cr. ratio on the 1st, 8th, 15th and 22nd day of life.

**Figure 2:**
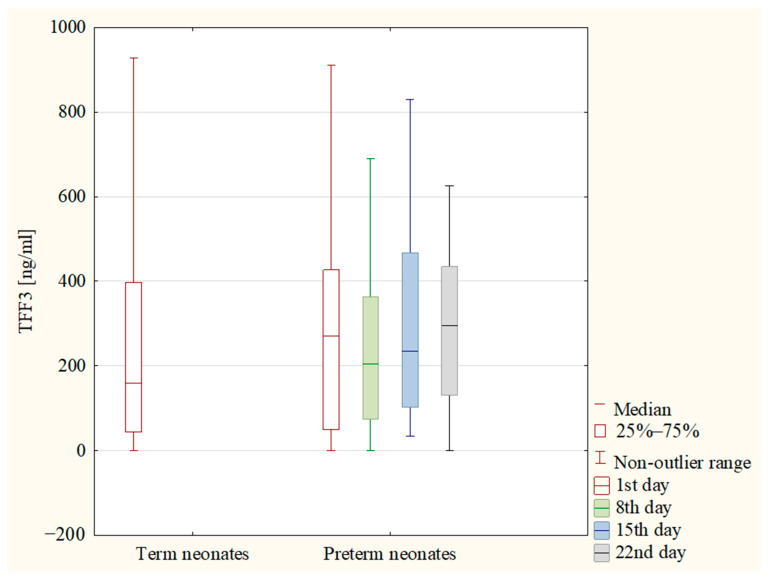
The concentrations of TFF3 in term neonates (1st day of life) and in preterm neonates (1st, 8th, 15th and 22nd day of life).

**Figure 3:**
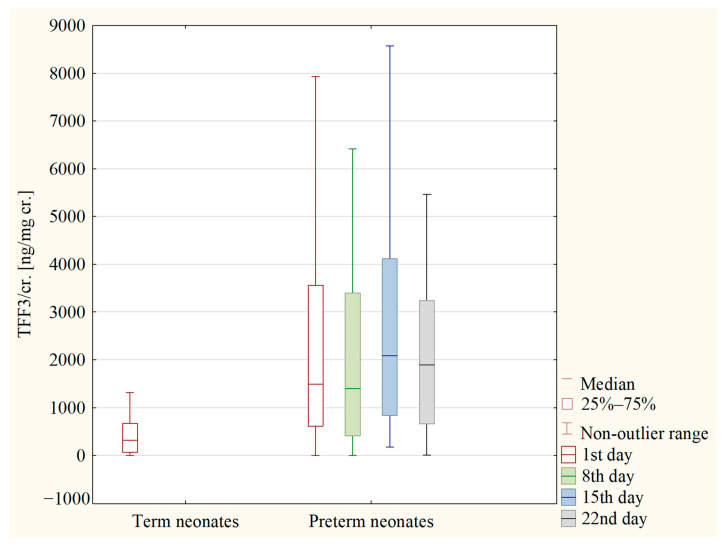
The values of TFF3/cr. ratio in term neonates (1st day of life) and in preterm neonates (1st, 8th, 15th and 22nd day of life).

The concentrations of TFF3 and the values of the TFF3/cr. ratio were analyzed on the following days of the preterm neonate’s life: 1st, 8th, 15th and 22nd. The statistical analysis showed no difference in the concentrations of TFF3 between the analyzed days. Also, the values of the TFF3/cr. ratio did not differ between these days. In Figure 2 and Figure 3, the concentrations of TFF3 and the values of the TFF3/cr. ratio in both the term and preterm neonates are presented.

The concentrations of TFF3 and the values of the TFF3/cr. ratio on each day of life were compared between the subgroups of preterm children based on sex, birth weight, type of delivery and gestational age. No differences in the above-mentioned parameters were found between the boys and girls, LBW and VLBW neonates, vaginal-born and cesarean-born children or very preterm and moderately + late preterm neonates.

The values of the eGFR, measured on the following days of the child’s life, showed a rising trend, being statistically higher on the 8th day than on the 1st day (*p* < 0.01), on the 15th day than on the 8th day (*p* < 0.01) and on the 22nd day than on the 15th day (*p* < 0.01).

### 3.6. The ROC Curve Analysis

**Figure 4:**
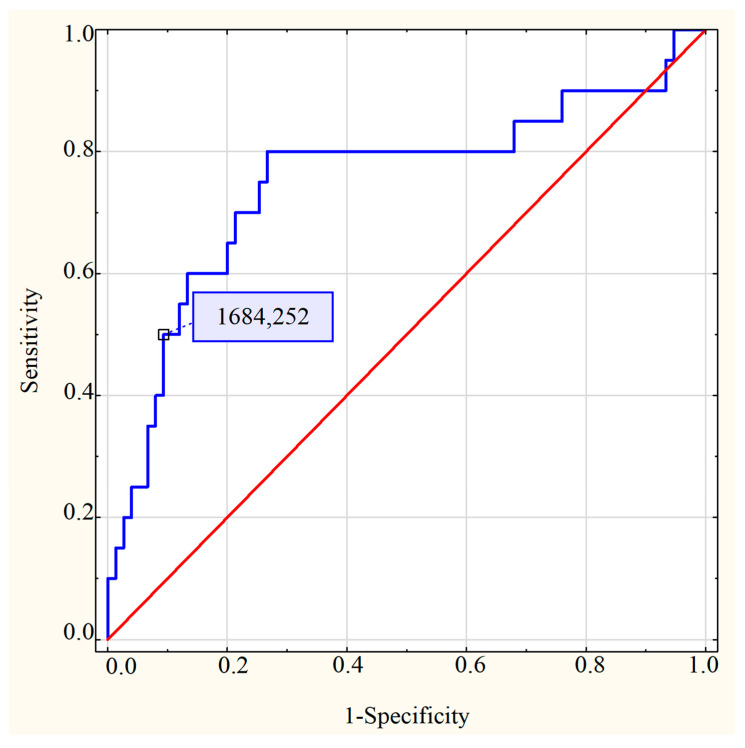
The ROC curve and the AUC of the TFF3/cr. ratio for the distinction between preterm and term neonates.

The ROC curve for the TFF3/cr. ratio in the preterm and term neonates showed AUC = 0.751 (95% CI 0.61–0.89) at a cut-off value of 1684.25 ng/mg cr. The sensitivity and specificity of the TFF3/cr. ratio to distinguish between the preterm and term neonates at the cut-off value were 50.00% and 90.70%, respectively (*p* < 0.01). The PPV was 58.82% and the NPV was 87.18%. The ROC curves were presented in the Figure 4.

## 4. Discussion

Kidney immaturity, a consequence of preterm delivery, is an important risk factor of tubular injury in premature neonates’ kidneys. This prospective study aimed to determine the values of urinary TFF3 in term and preterm neonates. It was hypothesized that TFF3 may be involved in the maturation and repair of premature kidneys.

The exact role of TFF3 in the kidneys remains uncertain. However, its functions are better understood in other tissues and organs. TFF3 plays a role in the restoration of intestinal epithelium and creates a barrier to protect it from injury. It inhibits the apoptosis of epithelial cells and promotes their survival and migration into lesions [15,16]. In the airways, TFF3 induces the differentiation of epithelial ciliated cells [17].

TFF3 is expressed in the tubular epithelial cells under pathological conditions, such as inflammation. Its expression may be stimulated by various cytokines (IL-4, IL-12, IL-6, TNF-α (tumor necrosis factor-α) and transcription factors (STAT3 (signal transducers and activators of transcription 3), HIF-1α (hypoxia-inducible factor-1α), C/EBPβ (CCAAT/enhancer binding protein), Sp1 (specificity protein 1), ETS (E26 transformation-specific) transcription factor) [18].

TFF3 acts as a mitogen, enabling cell migration and epithelial restitution. It separates the cell–cell and cell–substratum contacts and promotes the elongation of the epithelial cells and their migration to the injured, exfoliated surface. As the cell–cell contacts are mediated by E-cadherin (an adhesion molecule) binding to beta-catenin, the disruption in the complex comprising the above-mentioned molecules results in the loss of these contacts. To destroy the cell–cell adhesion, the phosphorylation of beta-catenin tyrosine is needed and TFF3 can induce this phosphorylation and promote the migration of the epithelial cells [19,20]. TFF3 is also involved in the expression’s regulation of other molecules (STAT3, Snail, Twist1) engaged in regulating E-cadherin expression [21,22]. In the case of the cell–substratum contacts, TFF3 impacts the matrix metalloproteinases (MMPs), which degrade the basemen membranes [23].

The epithelial cells are especially vulnerable to anoikis, the induction of apoptosis following the loss of the attachment to other cells and the extracellular matrix. For this reason, the antiapoptotic functions of TFF3 are important in the restitution of the epithelia [18]. TFF3 can regulate some of the most important apoptosis-related genes (Bcl-2 (B-cell lymphoma 2), Bax (Bcl-2-associated X protein), Bad (Bcl2 associated agonist of cell death)) and caspases. The most common of the final mediators is Bcl-2. TFF3 can regulate its expression by activating various signaling pathways (MAPK (mitogen activated protein kinase), NF-κB (nuclear factor κB), PI3K/AKT (phosphoinositide 3-kinase/protein kinase B) and STAT3 [18].

TFF3 can promote cells’ proliferation through the regulation of the signaling pathways involved in regulating the cell cycle [18]. Its pro-proliferation functions depend mainly on the PI3K/AKT signaling pathway [24]. The final stage is the inhibition of cyclin-dependent kinase inhibitors and an accumulation of cyclin D1, which together lead to disorders in the modulation of the cell cycle and the abnormal, increased proliferation of the cells [25,26,27]. Other pathways used by TFF3 to regulate cell proliferation are the Ras/Raf/MAPK pathway, started by EGF-R (epidermal growth factor receptor), and the Wnt pathway [15,28].

The angiogenic functions of TFF3 are based on its ability to upregulate HIF-1α (hypoxia-inducible factor-1α) and hypoxia-induced VEGF (vascular endothelial growth factor) synthesis, leading to neovascularization [16]. Also, the enhancement of IL-8 may play a role in this process, as IL-8 can promote the angiogenic responses of the endothelial cells [17].

The effects of TFF3 on the immune responses are still unclear. However, it was proven that TFF3 has anti-inflammatory activity, which depends on the protease activated receptor 2 (PAR-2) and the following signaling pathways: TLR (toll-like receptor)/NF-κB, PI3K/AKT, ERK (extracellular signal-regulated kinase)/Twist [18].

**Figure 5:**
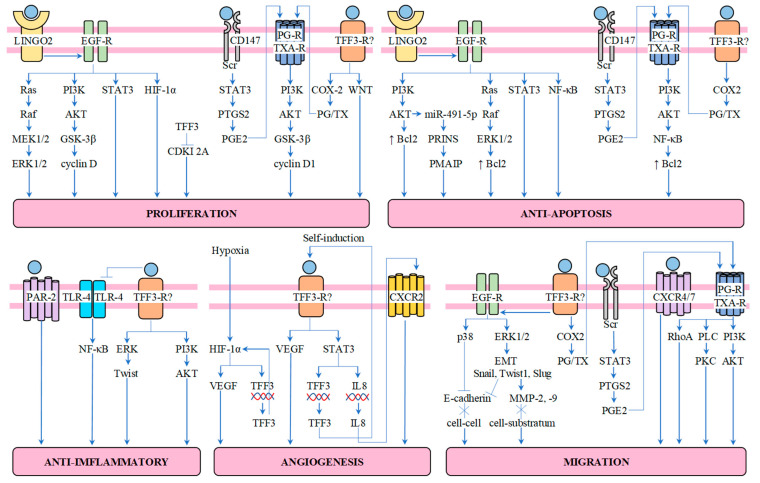
Schematic diagram of cell signaling mediated by TFF-3. The receptors for TFF3 are: LINGO2, PAR-2, CD147 and CXCR4/7. Binding of TFF3 to its receptor leads to: proliferation, survival, anti-inflammatory effects, angiogenesis and migration [18,29]. Abbreviations: TFF3—trefoil factor, LINGO2—leucine rich repeat receptor and nogo-interacting protein 2, EGF-R—epidermal growth factor receptor, PG-R—prostaglandin receptor; TXA-R—thromboxane A receptor; TFF3-R—TFF3 receptor, unknown; MEK—mitogen-activated protein kinase; ERK—extracellular signal-regulated protein kinase; PI3K—phosphoinositide-3-kinase; AKT—protein kinase B; GSK-3β—glycogen synthase kinase-3 beta; STAT3—signal transducers and activators of transcription; HIF-1α—hypoxia inducible factor 1α; CDKI 2A—cyclin-dependent kinase inhibitor 2A; PTGS2—prostaglandin-endoperoxide synthase 2; PGE2—prostaglandin E2; COX-2—cyclooxygenase-2; PG—prostaglandin; TX—thromboxane; WNT—wingless-related integration site; Bcl-2—B-cell lymphoma 2; PRINS—psoriasis susceptibility-related RNA gene induced by stress; PMAIP—phorbol-12-myristate-13-acetate-induced protein 1; NF-κB—nuclear factor κB; VEGF—vascular endothelial growth factor; IL8—interleukin 8; MMP—matrix metalloproteinases; PLC—phospholipase C; PKC—protein kinase C; RhoA—Ras homolog family member A; CXCR—C-X-C chemokine receptor; PAR-2—protease activated receptor 2; TLR—Toll-like receptor.

In the Figure 5. we tried to summarize the possible cell signaling pathways mediated by TFF3.

Nephrogenesis is a complex process that includes the interaction between the metanephric mesenchyme and the branching ureteric bud. The nephron progenitor differentiate and self-renew to form the nephron’s structures from the glomerulus to the collecting duct. The new nephrons are formed by the progenitor cells until 32–36 weeks of gestation. In preterm neonates, the nephrogenesis continues after birth [30]. Various molecular pathways are engaged in nephrogenesis, and some seem to coincide with the TFF3-mediated pathways. The differentiation of nephron progenitor cells, the morphogenesis of the ureteric bud and the renal endothelial migration and patterning all require, among other things, the activation of the RAS-MAPK and PI3K/AKT signaling pathway and beta-catenin, which promote the survival and proliferation of the nephron progenitor cells, ureteric bud cells and endothelial cells and promote vascular network formation. These molecular pathways are also activated by TFF3. In this case, it may be hypothesized that this molecule might take part in nephrogenesis. Also, the epidermal growth factor receptors (EGFR) take part in kidney development. It is important for the proliferation, differentiation, migration and growth of cells [31]. TFF3 is able to interact with LINGO-2 to regulate and enhance EGFR signaling [32]. This is further evidence of the possible involvement of TFF3 in nephrogenesis.

A study on mouse embryos showed the presence of TFF3 peptide in the developing kidney tubules during the later stages of kidney development. The TFF3 immunostaining was mostly visible in the cortical tubules, morphologically corresponding to both the proximal and distal convoluted tubules. It was also present in the medullary tubules, corresponding to the collecting loops and the thick limb of the Henle’s loop. Importantly, the glomeruli was negative for TFF3. The fact that TFF3 occurs in the later stages of kidney development suggests that it plays a role in the functional maturation of tubular cells and may be used as a marker of the maturation of the epithelium [33].

We still find many gaps in the roles of TFF3 in the kidneys. However, based on its properties, TFF3’s functions in the kidneys might be similar to its functions in the gastrointestinal tract and in the airways. It might take part in the repair of the renal tubular epithelium and its regeneration after the action of a damaging factor [34]. Because the expression of the TFF3 mRNA is highly associated with the excretion of TFF3 in urine, higher levels of TFF3 in the urine reflect its elevated secretion in the renal tubules and not its elevated filtration in the glomerulus [13]. Higher urinary concentrations of TFF3 might suggest ongoing repair in the tubules [35,36]. It may be concluded that TFF3’s roles in the restoration and regeneration of injured kidneys are underestimated and should be investigated not only because of the potential use of TFF3 as a biomarker of renal damage.

**Figure 6:**
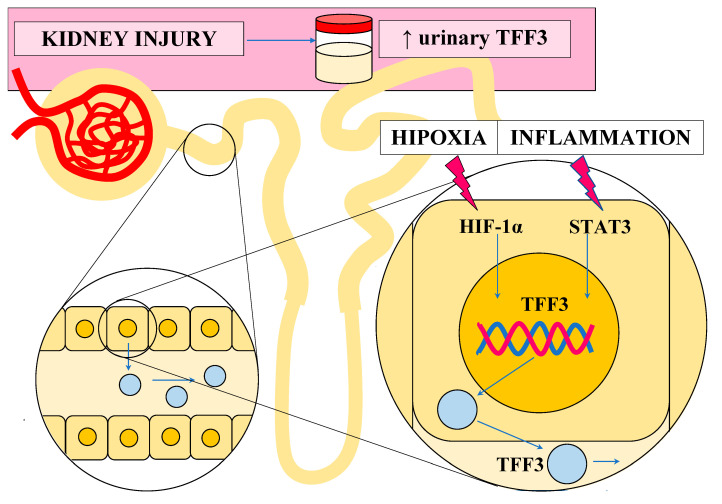
Potential explanation of the elevated expression of TFF3 during renal injury. Abbreviations: TFF3—trefoil factor 3; HIF-1α—hypoxia inducible factor 1α; STAT3—signal transducers and activators of transcription.

In the Figure 6. we tried to summarize the potential explanation of the elevated expression of TFF3 during renal injury.

This study showed that neonates excrete TFF3 in their urine. The value of the TFF3/cr. ratio in the preterm neonates was significantly higher than in the term neonates (median (Q1–Q3): 317.29 (68.07–671.40) and 1486.85 (614.92–3559.18) ng/mg cr., respectively). Unfortunately, in the literature, there are only a few studies concerning the urinary concentration of TFF3, and their results are inconsistent [12,37,38,39,40].

Yamanari et al. also studied adult patients with CDK. They analyzed the values of the TFF/cr. ratio in patients with early and late CDK ((median (Q1–Q3)): 45.6 (28.6–62.8) and 356.6 (237.4–462.7) ng/mg cr., respectively) [37]. Comparing these results with our results, the median value of the TFF3/cr. ratio in the term neonates was similar to that in patients with late CDK. The median value of the TFF3/cr. ratio in the preterm neonates was, however, significantly higher than in patients with CKD. Du et al. showed that the median values of the urinary TFF3/cr. ratio were positively correlated with the CDK stages, and were 367.1 ng/mg cr., 910.6 ng/mg cr., 1149.0 ng/mg cr., 1610.0 ng/mg cr. and 3475.0 ng/mg cr. for CKD stages 1–5, respectively [12]. Comparing these results with the results obtained during our study, the median values of the TFF3/cr. ratio in the term neonates were similar to the values in CDK stage 1, and the median values of this ratio in the preterm neonates showed similarity with the values in CDK stage 4. In an attempt to explain the elevated value of the TFF3/cr. ratio in the preterm children, it should be taken into consideration that the immaturity of the neonate’s kidney, especially the immaturity of the renal tubules and ongoing developmental changes that occur in the tubules, may lead to the elevated secretion of TFF3 and its excretion in the urine [41]. Also, perinatal stress, including oxidative stress, cannot be ignored as it may have a negative impact on the developing kidney [42]. Moreover, preterm birth itself can cause subclinical kidney damage [6]. The epithelial cells of the proximal tubules are prone to damage and TFF3 plays a role in the maintenance and repair of the epithelium [10,43].

In contrast, Askenazi et al., assessing a group of 111 neonates with birth weight ≤ 1200 g and/or gestational age ≤ 31 weeks, showed lower values of the TFF3/cr. ratio in both the group of neonates without AKI and the group with AKI. These values were (median (Q1–Q3)): 102 (69.9–249) and 100 (58.4–162) ng/mg cr., respectively [38].

Preterm neonates were also assessed by Correa et al. They showed that the urinary concentration of TFF3 in preterm neonates measured at 72 h of life was (median (Q1–Q3)): 0.022 (0.002–2.417) and at 3 weeks of life: 0.206 (0.003–26.388) ng/mL. These findings are very divergent and were unfortunately not normalized with the urinary creatinine concentration [39].

Much higher values of the TFF3/cr. ratio were shown by Brott et al. They examined 20 male and 19 female healthy adults aged 18–70 years and estimated a 95% CI for the value of TFF3/cr. to be 90.00–9060.00 ng/mg cr. [40]. The 95% CI for the value of the TFF3/cr. ratio measured for term neonates in our study was within the ranges proposed by the above-mentioned researchers. We did not assess the 95% CI for the preterm neonates, as the data were not normally distributed, and the group was too small to construct the confidence interval without the normal distribution.

No significant differences in both the concentrations of TFF3 and the values of the TFF3/cr. ratio were found between the subsequent days of the preterm neonates’ lives. An explanation of this phenomenon may be that in preterm neonates, nephrogenesis can continue during the first 40 days of life, and TFF3 may be engaged in proliferation and angiogenesis. An additional explanation may be related to the fact that, during this time, the kidneys are highly vulnerable to injury and TFF3 takes part in the processes of repair [7,44].

In the group of term neonates, both the concentrations of TFF3 in the urine and the values of the TFF3/cr. ratio were significantly higher in boys than in girls. However, the explanation of this phenomenon is not known. In the group of preterm neonates, no significant differences in the above-mentioned parameters were found between the boys and girls. It is possible that the number of preterm neonates was too small to assess these differences.

## 5. Limitations

The study has a relatively small number of patients, especially in the group of preterm neonates. The small number of preterm neonates resulted from the strict inclusion criteria (only healthy neonates). The child had to be hospitalized for at least four weeks and had to have four urine samples collected in the subsequent days of life. Because of the small size of the group comprising preterm neonates, it was difficult to determine the actual impact of the gestational age (very preterm vs moderately and late preterm) and the birth weight (low and very low birth weight) on the concentration of TFF3 in the urine of the preterm neonates. The obtained data showed no significant difference in the concentration of TFF3 and the value of the TFF/cr. ratio between the above-mentioned subgroups of preterm children. However, studies on larger groups of children would be valuable to confirm this observation.

The eGFR values were not calculated in the term neonates. The routine practice in the department does not require taking blood samples from healthy neonates and measuring the serum concentration of creatinine.

The study was concentrated on the urinary levels of TFF3. It may also be valuable to measure the urinary concentration of TFF3 in parallel with assessing the serum levels of TFF3. This would provide a better understanding of TFF3 handling in the human body.

We conducted the study on samples. To make the sample representative for the population of healthy term neonates or the population of stable preterm neonates, we assessed all of the children who were born during the study and who met the inclusion criteria without meeting the exclusion criteria. However, taking into consideration the small number of children who were assessed and the strict criteria used to make the study group, the results cannot be generalized to other populations. It was a preliminary study and the observed associations and observations should be explored in other, larger studies. It would be valuable to conduct similar studies considering term and preterm neonates in different clinical conditions. Extending the study would provide a better understanding of both the secretion and excretion of TFF3. This would be valuable for future biomarker qualification steps. Moreover, it would answer the question of whether the findings could be generalized to patients with different characteristics than the characteristic included in our study.

## 6. Conclusions

Prematurity is associated with increased urinary excretion of TFF3. The excretion of TFF3 on the 1st day of life was significantly higher in the preterm neonates than in the term neonates. In the premature neonates, the urinary excretion of TFF3 did not change significantly during the first 4 weeks of the neonate’s life. Male gender is associated with an increased urinary TFF3 excretion in term neonates.

## Figures and Tables

**Table 1:** Characteristics of the preterm neonates.

	Preterm Neonates (*N* = 20)	Boys (*N* = 8)	Girls (*N* = 12)	*p*
	Median (Q1–Q3)			
Gestational age (weeks)	30.5 (29.5–32.5)	32.0 (30.5–33.5)	30.0 (28.5–31.5)	<0.05
Very preterm/moderately + late preterm	13/7	4/4	9/3	NS
Vaginal/cesarean delivery	6/14	2/6	4/8	NS
Birth weight (g)	1600 (1220–1875)	1875 (1600–2055)	1305 (1200–1680)	<0.05
LBW/VLBW/ELBW	11/8/1	7/1/0	4/7/1	<0.05
Length (cm)	45.00 (38.50–47.00)	46.00 (45.00–47.00)	41.00 (37.50–46.50)	NS
Head circuit (cm)	29.00 (27.50–30.50)	30.00 (29.00–32.00)	29.00 (26.50–30.00)	NS

*p*–comparison of boys and girls; NS—non statistical; ELBW—extremely low birth weight; LBW—low birth weight; VLBW—very low birth weight.

**Table 2:** Concentrations of TFF3 and the values of TFF3/cr. ratio on the 1st day of life.

Parameters	All Neonates	Boys	Girls	*p*
	Median (Q1–Q3)			
Term neonates				
TFF3 (ng/mL)	159.94 (43.65–397.84)	339.46 (157.79–726.12)	74.85 (11.08–200.17)	<0.01
TFF3/cr. (ng/mg cr.)	317.29 (68.07–671.40)	427.16 (210.26–975.10)	125.73 (19.02–361.47)	<0.01
Preterm neonates				
TFF3 (ng/mL)	271.50 (49.60–426.72)	332.43 (77.08–435.48)	186.14 (34.55–375.62)	NS
TFF3/cr. (ng/mg cr.)	1486.85 (614.92–3559.18)	1818.87 (849.36–3649.62)	1205.63 (358.83–3253.80)	NS

TFF3—trefoil factor 3; cr.—creatinine; *p*—comparison of boys and girls; NS—non statistical.

**Table 3:** Concentrations of TFF3 and the values of TFF3/cr. ratio on the 1st, 8th, 15th and 22nd day of life.

	All Preterm Neonates (*N* = 20)	Boys (*N* = 8)	Girls (*N* = 12)	*p*
	Median (Q1–Q3)			
1st day of life				
TFF3 (ng/mL)	271.49 (49.60–426.72)	332.42 (77.08–435.48)	186.14 (34.55–375.62)	NS
TFF3/cr. (ng/mg cr.)	1486.85 (614.92–3559.18)	1818.87 (849.36–3649.62)	1205.63 (358.83–3253.79)	NS
8th day of life				
TFF3 (ng/mL)	203.67 (73.32–363.69)	134.82 (37.16–233.47)	277.37 (153.19–395.99)	NS
TFF3/cr. (ng/mg cr.)	1399.90 (409.72–3398.79)	728.80 (277.27–1362.90)	2514.94 (810.41–4722.92)	NS
15th day of life				
TFF3 (ng/mL)	233.96 (101.74–466.86)	169.50 (122.65–547.06)	267.57 (77.23–466.86)	NS
TFF3/cr. (ng/mg cr.)	2086.65 (836.55–4112.48)	1438.18 (741.38–4351.44)	2694.93 (863.78–3870.29)	NS
22nd day of life				
TFF3 (ng/mL)	294.45 (131.06–433.98)	261.52 (156.22–372.70)	326.19 (131.06–608.66)	NS
TFF3/cr. (ng/mg cr.)	1891.45 (661.49–3241.30)	2402.01 (1084.64–4450.19)	1614.23 (464.76–2587.89)	NS

TFF3—trefoil factor 3; cr.—creatinine; *p*—comparison of boys and girls; NS—non statistical.

## Data Availability

Not applicable.
